# Analysis of Potential Hub Genes for Neuropathic Pain Based on Differential Expression in Rat Models

**DOI:** 10.1155/2022/6571987

**Published:** 2022-03-03

**Authors:** Jie Bai, Bin Geng, Xingwen Wang, Shenghong Wang, Yayi Xia

**Affiliations:** ^1^Department of Anesthesiology, Lanzhou University Second Hospital, Lanzhou, Gansu, China; ^2^Orthopedics Key Laboratory of Gansu Province, Lanzhou, Gansu, China; ^3^Department of Orthopaedics, Lanzhou University Second Hospital, Lanzhou, Gansu, China

## Abstract

**Objective:**

Neuropathic pain (NP) is a type of intractable chronic pain with complicated etiology. The exact molecular mechanism underlying NP remains unclear. In this study, we searched for molecular biomarkers of NP.

**Methods:**

Differentially expressed genes (DEGs) were predicted by analyzing three NP-related microarray datasets in Gene Expression Omnibus with robust rank aggregation. A weighted gene coexpression network analysis was conducted to construct a network of differentially expressed genes, followed by the evaluation of correlations between gene sets and the determination of hub genes. The candidate genes from the key module were identified using a gene set enrichment analysis.

**Results:**

In total, 353 upregulated and 383 downregulated genes were obtained, among which five hub genes were determined to be related to pain phenotypes. Reverse transcription-quantitative polymerase chain reaction was performed to verify the expression of these hub genes in the dorsal root ganglia of rats with spared nerve injury, which revealed the decreased expression of EMC4. Hence, EMC4 was defined as a biomarker for NP development.

**Conclusions:**

The results of this study form a basis for further research into the mechanism of NP development and are expected to aid in the development of novel therapeutic strategies.

## 1. Introduction

As a type of chronic pain with complex etiology, neuropathic pain (NP) is characterized by hyperalgesia, numbness, and allodynia [1]. More than 6% of patients experience debilitating NP-related physical and emotional trauma. Damage to the sensory system afflicts the transmission of sensory signals, thus resulting in hyperalgesia symptoms [2]. Owing to the complex pathogenesis of NP, there is still no effective treatment. With the development of bioinformatics, gene expression datasets such as those in the Gene Expression Omnibus (GEO) have been widely utilized to construct genetic networks and identify the potential roles and functions of differentially expressed genes (DEGs) in the development of NP [3].

However, the study of molecular mechanisms has significant implications in the treatment of NP. Dorsal root ganglia (DRGs) have previously attracted the attention of researchers. DRGs consist of pseudounipolar neurons that transmit signals from the peripheral nerves to the dorsal horns via the neuronal cell bodies [4]. Evidence shows that genetic variations in DRGs are related to pain phenotypes [5]. Therefore, it is of importance to analyze gene expression changes in the DRGs after peripheral nerve injury for the understanding of the molecular mechanism underlying NP, which may contribute to the development of an effective therapeutic regimen.

A large number of gene studies on the molecular basis of NP have been performed, but most are based on the analysis of peripheral blood samples from patients to identify DEGs. Therefore, the non-spinal cord in situ genetic information obtained does not truly reflect the lesion site. Additionally, it is difficult to obtain a stable and reproducible phenotype owing to the complex background of patient samples [6].

Some investigators have utilized rat models with more stable phenotypes to map genetic changes at the site of NP lesions. For example, Yu H et al. screened for hub genes and measured the expression of these genes in a rat model of NP. As a result, the authors identified DEGs substantially enriched in “extracellular space,” and their Kyoto Encyclopedia of Genes and Genomes (KEGG) pathway enrichment analysis showed that DEGs were enriched in inflammatory disease and mitogen-activated protein kinase signaling pathways [7]. However, these findings were not assessed in rat models for further validation, and thus, there is still a lack of reliable analyses of molecular mechanisms and hub genes in NP.

In this context, we proposed a research hypothesis that potential core genes for NP could be identified by DEGs and replicated in animal models. This work was conducted to detect the expression of DEGs and determine their reproducibility in an animal model of NP. Because in situ information could not be obtained from human samples, a phenotypically stable and reproducible animal model was employed to ascertain the pathogenesis of NP. Our results provide novel insights into central genes and biological pathways in the pathogenesis of NP using bioinformatics analyses.

## 2. Materials and Methods

### 2.1. Selection of Gene Microarray Datasets

Microarray data were downloaded from the GEO database (http://www.ncbi.nlm.nih.gov/geo/) for expression profiling of neuralgia. Three independent microarray datasets, including GSE24982, GSE30691, and GSE63442, were selected. GSE24982 comprised data from 20 spinal nerve ligation-induced NP model animals and 20 sham cohort samples generated using the GPL1355 microarray platform (Affymetrix Rat Genome 230 2.0 Array). GSE30691 included data from 56 samples of three independent NP models, which were generated using the GPL85 microarray platform (Affymetrix Rat Genome U34 Array). GSE63442 consisted of data from six spinal nerve ligation model animals and six sham control samples produced using the GPL341 microarray platform (Affymetrix Rat Expression 230A Array). Raw data were preprocessed using *R* software (version 4.0.2; http://www.R-project.org/).

### 2.2. Identification of DEGs

Analysis of DEGs was carried out to retrieve genes with differential expression in the treatment group compared to the control group. Microarray data were normalized to predict DEGs between the control and treatment groups using the Limma package [8]. Robust rank aggregation (RRA) was applied to identify reliable DEGs, minimize inconsistencies, and integrate the results of the three datasets. Firstly, the genes in each dataset were ranked as per their fold-changes among the groups. Then, the “RRA” package was utilized to integrate the list of all ranked genes. The adjusted *P* value of all genes was obtained to indicate the possibility of their ranking in the resultant gene list. DEGs were screened out with the threshold of log2 (fold-change) >0.3 and adjusted *P* < 0.05 as screening criteria, followed by the construction of a new data frame. Finally, a “pheatmap” package in *R* was used to visualize and plot the top 40 DEGs (the top 20 upregulated and downregulated genes were selected based on the adjusted *P* value).

### 2.3. Functional Enrichment Analyses

Methods and tools have been developed to analyze and filter these datasets to produce smaller, more meaningful, and biologically relevant gene/protein lists. The functional enrichment analysis is an approach that can identify genes enriched in the datasets of molecular functions, biological processes, and pathways of interest. Functional enrichment analysis contributes to the focus of researchers on a specific gene of interest or a specific biological problem. Considering a threshold of *P* < 0.05, the clusterProfiler package [9] was applied for Gene Ontology (GO) and KEGG pathway analyses. Then, the enrichment results were visualized using the GOplot package [10] to further analyze the biological functions of genes related to NP.

### 2.4. Identification and Verification of Hub Genes

To identify the modules most closely correlated with NP phenotypic traits, a weighted gene coexpression network analysis (WGCNA) was performed to construct a scale-free network, define a coexpression matrix and adjacency functions, and calculate the coefficients of different nodes. The GSE24982 dataset was selected for the identification of coexpression modules. Based on the RRA analysis results, the top 4000 genes (according to the *P* value) from GSE24982 were extracted, followed by WGCNA of expression data to obtain the key modules with the highest correlation with NP. Modules and candidate genes were acquired using the WGCNA package [11]. An unsigned topological overlap matrix was employed to construct the WGCNA network and determine coexpressed gene modules. The soft-thresholding power was set to 12 with the threshold for cutting height as 0.25, and the minimum number of gene modules as 30. The degree of correlation between genes and modules was determined as module membership (MM), and the degree of correlation between genes and clinical information was regarded as gene significance (GS). Genes with high connectivity tended to have crucial functions. Therefore, genes with high correlation were defined as hub genes in the candidate modules. The hub genes in the modules met the criteria of MM > 0.80 and GS > 0.70. The top 50 genes with the highest connectivity were selected to confirm the expression data in GSE63442 and GSE30691 using an independent *t*-test.

### 2.5. Gene Set Enrichment Analysis (GSEA) and Gene Set Variation Analysis (GSVA)

GSEA and GSVA refer to the analyses based on gene sets. As the name implies, a gene set is a collection of genes, and any number of genes together can be called a gene set. However, the gene set applied for analyses must have a certain biological significance. The most widely used gene set databases are GO and KEGG, one of which classifies genes according to GO, and the other integrates related genes as per metabolic pathways. In addition, genes can be assembled into biologically significant gene sets through transcription factor regulatory networks, coexpression networks, and lists of marker genes that define biological states. The *R* packages “clusterProfiler” and “GSVA” were employed to perform GSEA on candidate hub genes to yield the biological pathways related to these genes. The six samples in GSE63442 were grouped according to the median expression of the candidate hub genes: high and low expression groups. *P* < 0.05 was considered to be statistically significant.

### 2.6. Animal Experiments

Our study was ratified and reviewed by the Animal Care and Use Committee of the Second Hospital of Lanzhou University (D2019-003). Twelve adult male Sprague Dawley rats (weighing 200–220 g) were purchased from the Lanzhou Institute of Veterinary Medicine, Chinese Academy of Agricultural Sciences. There were 2 groups in this study, spared nerve injury group (SNI, *n* = 6) and sham-operated group (Sham, *n* = 6). Animals were housed in cages with a 12-h light/dark cycle and food and water *ad libitum*.

### 2.7. Establishment of a Rat Model of NP

To establish an NP model, spared nerve injury (SNI) was induced in the left sciatic nerve of rats following a previously published protocol [12]. Briefly, after intraperitoneal injection with sodium pentobarbital (40 mg/kg), the skin of the left lateral thigh was incised, and the biceps femoris muscle was bluntly dissected to expose the terminal branches of the sciatic nerve. At the bifurcation point, the common peroneal nerve and tibial nerve were tightly ligated with 4–0 silk that was then severed at the distal end of the ligature. Approximately 3–5 mm of the distal end of the nerve was removed, and contact with the sural nerve was avoided during the surgery. Following the confirmation of complete hemostasis, the wound was sutured. In sham-operated rats, the sciatic nerve was exposed without ligation and incision. Six rats each were utilized as biological replicates in the Sham and SNI groups. The sample size was not predetermined based on a priori power calculations but was estimated based on previous literature and the authors' experience.

### 2.8. Measurement of Mechanical Hyperalgesia

Von Frey filaments (Aesthesio, Danmic Global, USA) were adopted to assess the withdrawal threshold for mechanical hyperalgesia in rats as previously described [13]. Rats were placed in transparent boxes with a wire mesh platform to acclimatize for at least 30 min. Then, the lateral edge in the left hind paw of the rats was stimulated with von Frey filaments, and the stimulation intensity started from 2 g. The paw withdrawal threshold (PWT) was determined using the up-and-down method described by Chaplan et al. [13]. The measurement was repeated three times for each rat, and the response frequency of each filament force was recorded and expressed as a percentage. The mechanical withdrawal threshold of all rats was tested 3 days before surgery and 3, 7, 10, and 14 days after surgery. On day 14 after SNI surgery, all animals were euthanized by administering an overdose of sodium pentobarbital after the assessment of mechanical hyperalgesia. Then, the histological samples were collected.

### 2.9. Quantitative Real-Time Polymerase Chain Reaction (RT-qPCR) Analysis

L_3-5_ DRG neurons were collected and frozen in liquid nitrogen after SNI or sham surgery (*n* = 6 in each group). Total RNA was extracted using a TRIzol reagent (Invitrogen, Carlsbad, USA) following the manufacturer's protocols. SYBR Premix Ex Taq^TM^ was applied for qPCR. Gene expression was calculated using the 2^–△△CT^ method with glyceraldehyde-3-phosphate dehydrogenase (GAPDH) as a normalizer. The primers used for qPCR are listed in Table 1. The ggstatsplot package was used to perform *t*-test and created graphics.

### 2.10. Statistical Analysis

GraphPad Prism 8 was employed for statistical analysis. The data are summarized as the mean ± standard deviation, and the Shapiro–Wilk test was used to examine the normal distribution of data. The sample size was not predetermined based on a priori power calculations [14]. It was estimated based on previous literature and the authors' experience. Repeated measures analysis of variance (ANOVA) (factors: group and time) was utilized to compare PWT evoked by mechanical stimulus between ipsilateral and contralateral paw of rats in the Sham and SNI groups. Student's *t*-test was carried out to compare the expression of five hub genes between the Sham and SNI groups. A *P* value < 0.05 was considered statistically significant. *P* value was adjusted based on Benjamini–Hochberg FDR using the *R* package fdrtool.

## 3. Results

### 3.1. Identification of DEGs

**Figure 1:**
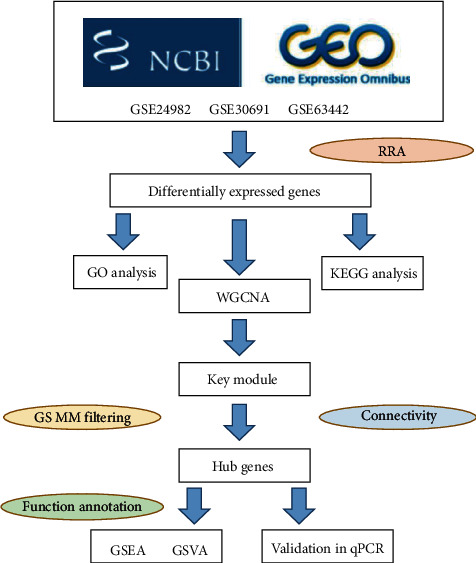
The workflow of our study. GEO, Gene Expression Omnibus; GO, Gene Ontology; KEGG, Kyoto Encyclopedia of Genes and Genomes; WGCNA, weighted gene coexpression network analysis; GS, gene significance; MM, module membership; GSEA, gene set enrichment analysis; GSVA, gene set variation analysis.

Figure 1 depicts the process flow of identification and functional analyses of DEGs. Three datasets in the GEO database were used for RRA analysis, which integrated DEGs from three GSE datasets and retrieved intersected DEGs. According to the RRA analysis results, 353 upregulated and 383 downregulated genes were filtered out based on a threshold of *P* < 0.05 (Supplementary 1). The most significantly upregulated gene was REG3B (*P* = 3.85*E* − 07 and adjusted *P* = 2.96*E* − 03), followed by ATF3 (*P* = 4.33E-07 and adjusted *P* = 2.96E-03). The most significantly downregulated gene was DDYSL4 (*P* = 5.94*E* − 08 and adjusted *P* = 8.12E-04), followed by KCNS3 (*P* = 1.92E-07 and adjusted *P* = 1.31E-03).

**Figure 2:**
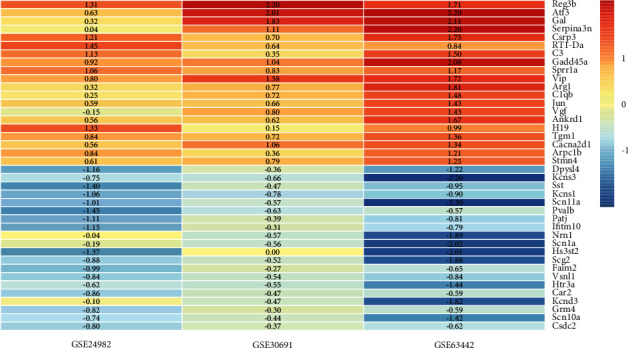
The heatmap of the top 20 upregulated and downregulated genes identified by RRA analysis. Each column represented a dataset, and each row represented one gene. Red represented upregulated genes, while blue represented downregulated genes. The Limma R package was utilized to calculate the logarithmic fold change of each dataset, which was expressed with the numbers in the heatmap. DEG, differentially expressed gene; RRA, robust rank aggregation.

The top 20 upregulated and downregulated genes are shown in a heatmap in Figure 2, where the specific FDR-adjusted *P* value for each gene is shown in Supplementary 4. Following RRA analysis, a PPI network was constructed to define the relationship between the proteins expressed by the DEGs (Supplementary Figure 1), which increases our understanding of protein functions and relationships. Research has extensively explored the significant roles of some genes identified, such as REG3B, ATF3, SPRR1A, GAL, and JUN, in NP.

### 3.2. Functional Enrichment Analyses of DEGs

**Figure 3:**
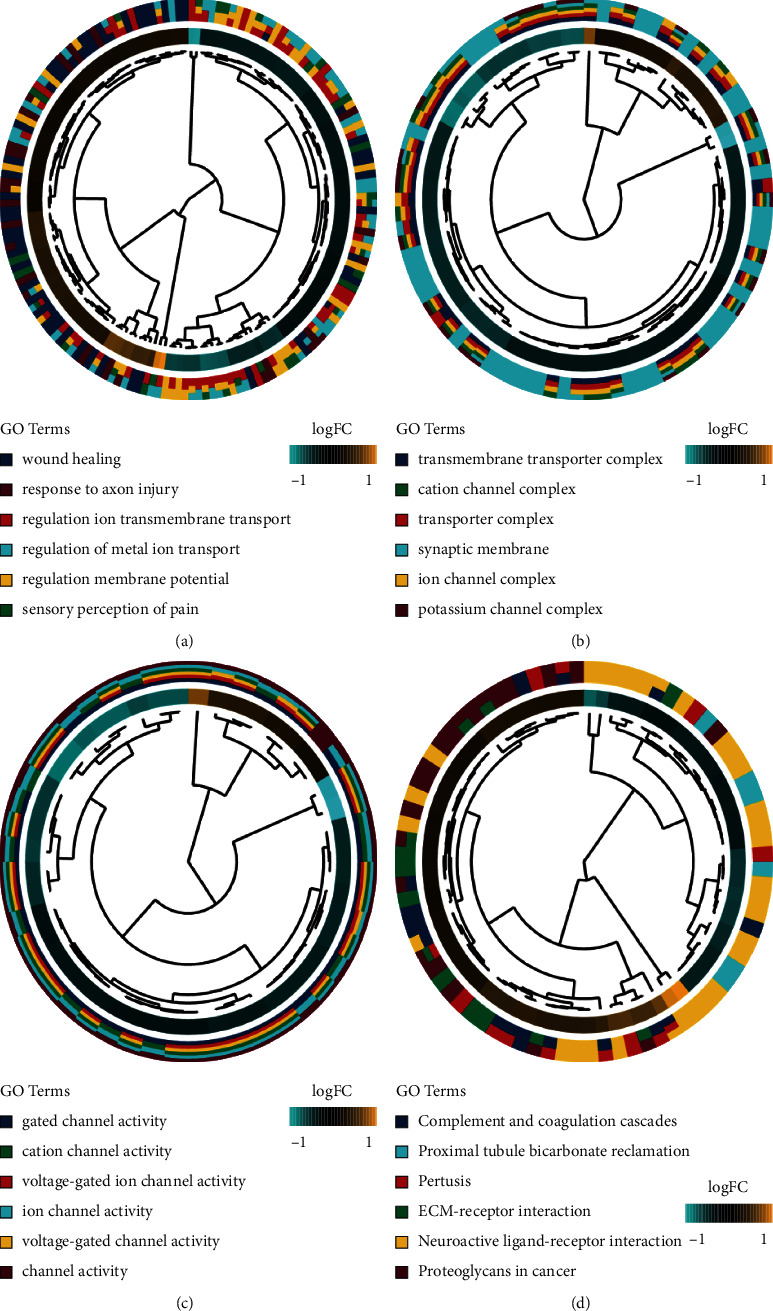
The GO and KEGG analyses of the DEGs identified by the RRA analysis. (A-C) The Chord plot of GO enrichment analysis of the DEGs in three parts: biological process (BP), cellular component (CC), and molecular function (MF). (D) The Chord plot of KEGG pathways enrichment analysis of the DEGs. GO, Gene Ontology; KEGG, Kyoto Encyclopedia of Genes and Genomes; RRA, robust rank aggregation.

The DEGs identified by RRA analysis were subjected to GO and KEGG functional enrichment analyses. “Regulation of ion transmembrane transport,” “sensory perception of pain,” “response to axon injury,” “regulation of membrane potential,” and “potassium ion transport” were the most substantially activated biological functions of the DEGs (Figures 3(a)–3(c)). KEGG analysis showed that the DEGs were remarkably enriched in 27 signaling pathways, including “complement and coagulation cascade,” “neuroactive ligand-receptor interaction,” and “ECM-receptor interaction” (Figure 3(d)). The pathways identified are closely related to the development of NP, suggesting that the obtained DEGs are indeed related to NP.

### 3.3. Identification and Validation of Hub Genes

**Figure 4:**
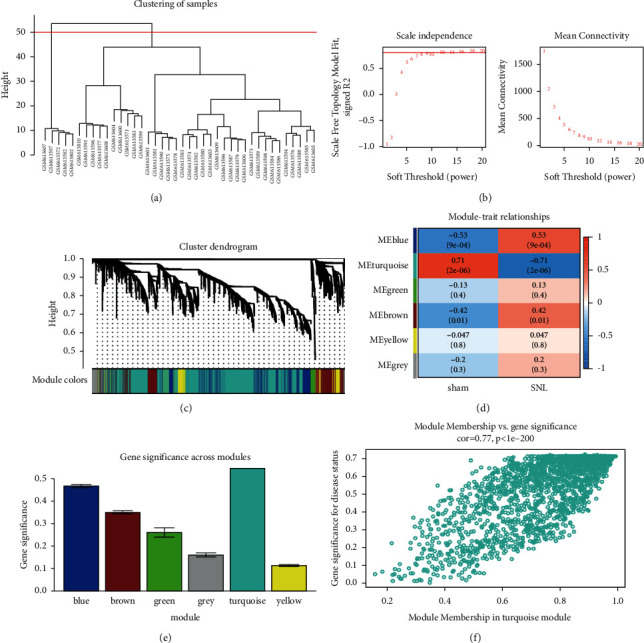
WGCNA. (a) The Cluster dendrogram of the module eigengenes. (b) The selection of soft-thresholding powers through scale-free topology fit index and mean connectivity among genes. (c) The cluster dendrogram of all DEGs based on the dissimilarity measure and the assignment modules. (d) The heatmap of module-trait correlations. The number in each small box represented the corresponding correlation and *P* value. (e) The identification of modules related to NP. The horizontal axis indicated the name of modules, and the vertical axis indicated the gene significance value. (f) The scatter plot of GS and MM for genes in the turquoise module. DEG, differentially expressed gene; WGCNA, weighted gene coexpression network analysis.

**Figure 5:**
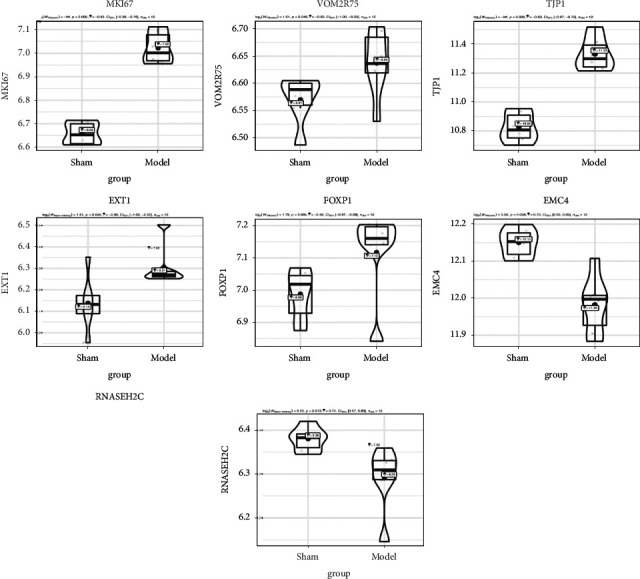
The selection and verification of the candidate hub genes. The expression of MKI67, VOM2R75, TJP1, EXT1, FOXP1, EMC4, and RNASEH2C differed between the two groups. The genes with the highest connectivity were screened out by WGCNA. To validate the expression data of these genes, the GSE63442 and GSE30691 datasets were selected for pairwise validation by independent *t*-test. The ggstatsplot package was used to perform *t*-test and plot graphs. GEO, Gene Expression Omnibus. Statistical analysis was conducted using the independent *t*-test. Plots represented mean ± 95% confidence interval (CI).

A hub gene is a gene that plays a critical part in a biological proc gene. WGCNA was adopted to investigate functional modules and genes associated with clinical traits. The key modules were identified by setting the soft-thresholding power as 12, the cutting height as 0.25, and the minimum module size as 30 followed by the determination of six modules with sizes from 77 to 2209 genes (labeled with different colors in Figure 4). The correlations between module eigengenes and clinical traits are visualized in Figures 4(c)–4(e). From the heatmap of module–trait relationships, it was found that the turquoise module, which contained 2, 209 genes, shared the strongest correlation with clinical traits (Figures 4(d) and 4(f)). Based on a heatmap of module–feature correlations, the turquoise module was considered the key module (correlation coefficient = 0.71 and *P* = 2*E* − 06; Figure 4(d)). The enrichment of DEGs of the turquoise module in GO and KEGG pathways is shown in Supplementary 3. As shown in Figure 4(d), genes in the blue module (correlation coefficient = 0.53 and *P* = 9*E* − 04) were also correlated with NP traits. The top 50 genes with the highest connectivity (Supplementary 2) were extracted based on the screening criteria for candidate hub genes in the key modules (MM > 0.80 and GS > 0.70). Seven genes (MKI67, VOM2R75, TJP1, EXT1, FOXP1, RNASEH2C, and EMC4) were retrieved as candidate hub genes. Among these, five genes were upregulated, whereas only RNASEH2C and EMC4 were downregulated in the NP group (Figure 5).

### 3.4. Screening of the Five Hub Genes by GSEA and GSVA

**Figure 6:**
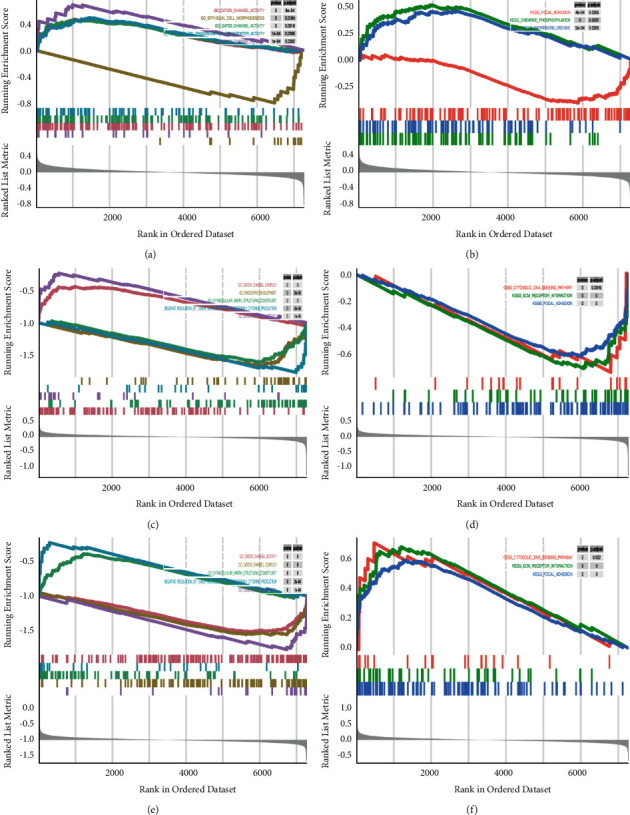
The GSEA of the candidate hub genes in the GEO dataset. (A) C, (E) GSEA of the single candidate hub genes in GO terms according to the normalized enrichment scores. (C) MKI67, (G) EMC4, (M) RNASEH2C. (B) D, (F) GSEA of the single candidate hub genes in the KEGG pathway. (D) MKI67, (H) EMC4, (N) RNASEH2C. GEO, Gene Expression Omnibus; GO, Gene Ontology; GSEA, gene set enrichment analysis.

**Figure 7:**
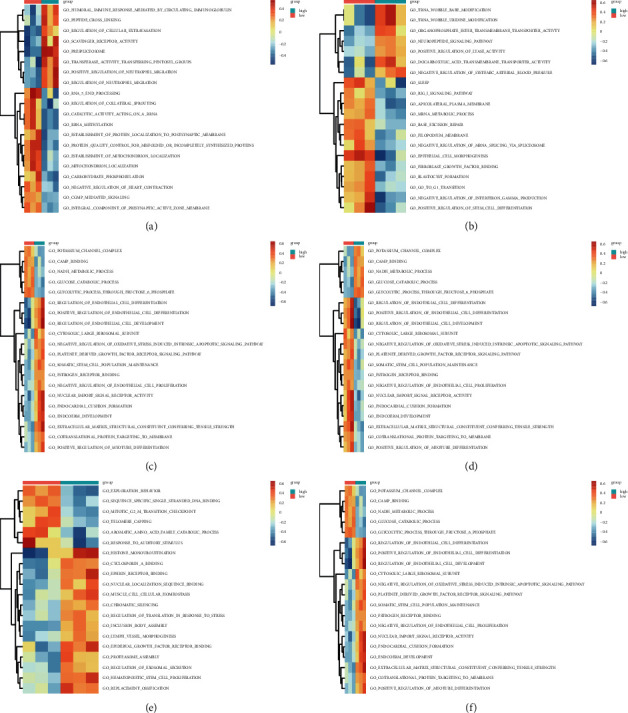
The GSVA of the candidate hub genes. There existed GSVA-derived clustering heatmaps between the single candidate hub genes and the GO terms. Only signaling pathways with log(fold change) > 0.2 are presented. (a) VOM2R75, (b) MKI67, (c) TJP1, (d) EMC4, (e) FOXP1, (f) RNASEH2C. GEO, Gene Expression Omnibus; GO, Gene Ontology; GSVA, gene set variation analysis.

To ascertain the biological functions of the seven candidate hub genes involved in NP development, GSEA and GSVA were conducted on the GSE63442 dataset. As exhibited in Figures 6 and 7, and Supplementary Figure 2, the genes in the groups with high expression of MKI67, TJP1, EXT1, RNASEH2C, or EMC4 were enriched in “sodium channel activity” in accordance with the GO term enrichment analysis. The genes of the MKI67, TJP1, RNASEH2C, and EMC4 high-expression groups were enriched in “cation channel activity” and “cation channel complex.” Further, TJP1, RNASEH2C, and EMC4 were all enriched in the pathways “cytosolic DNA sensing pathway,” “ECM-receptor interaction,” and “focal adhesion.” Therefore, these five hub genes (MKI67, TJP1, EXT1, RNASEH2C, and EMC4) were selected for further verification.

### 3.5. Behavioral Changes of Rats following SNI

**Figure 8:**
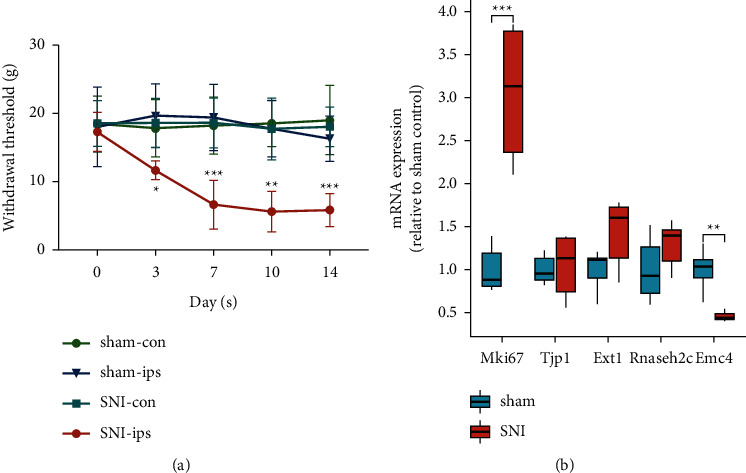
PWT changes after SNI surgery in rats and experimental validation of the expression of hub genes. (a) Compared with the sham-operated rats, the PWT of SNI rats decreased from day 3 after surgery. The withdrawal threshold was evaluated by von Frey filaments as a response evoked by a mechanical stimulus over time. The data in the sham and SNI groups were normally distributed. Repeated measures ANOVA followed by Tukey's multiple comparisons test was used to evaluate mechanical hyperalgesia, and data were expressed as mean ± standard deviation.  ^*∗*^, *P* < 0.05,  ^*∗∗*^, *P* < 0.01,  ^*∗∗∗*^, *P* < 0.001, compared with the sham-ips group on the corresponding days. (b) The expression of the five hub genes in DRG tissue after SNI surgery was validated by RT-qPCR. The data in the two groups were normally distributed. Unpaired Student's (t) test was carried out, and the data were expressed as mean ± standard deviation.  ^*∗∗*^, *P* < 0.01,  ^*∗∗∗*^, *P* < 0.001 compared with the Sham group. DRG, dorsal root ganglia; SNI, spared nerve injury; PWT, paw withdrawal threshold.

Peripheral nerve injury can cause hyperalgesia and allodynia in affected rats [15]. Following SNI, the rats developed mechanical allodynia-like behavior, which was evidenced by the reduction of the ipsilateral withdrawal threshold from days 3 to 14 after injury (*n* = 6, male) (Figure 8(a), Table 2 and Supplementary 5). The ipsilateral withdrawal threshold of rats in the SNI group exhibited conspicuously lower values than that in the sham group (3 days, *P* = 0.045; 10 days, *P*= 0.008; 7, 14 days, *P* < 0.001). From preoperative stage to 14 days after injury, there were no apparent differences between the Sham and SNI groups in terms of the withdrawal threshold of the contralateral paw (3 days, *P* = 0.965; 7 days, *P* = 0.992; 10 days, *P* = 0.922; 14 days, *P* = 0.783). The results illustrate the successful establishment of the NP rat model.

### 3.6. Validation of Hub Genes Using RT-qPCR

RT-qPCR was performed to measure the mRNA levels of the five hub genes. Compared with the sham-operated rats, MKI67 expression increased (*P* = 0.001; Figure 8(b)), and EMC4 expression clearly decreased in rats after SNI surgery (*P* = 0.002; Figure 8(b), Table 3 and Supplementary 6). These results are consistent with those of the microarray analyses. However, the expression of the other genes was not appreciably different between the SNI and sham-operated rats (TJP1, *P* = 0.793; EXT1, *P* = 0.053; RNASEH2C, *P* = 0.151; Figure 8(b), Table 3, and Supplementary 6).

## 4. Discussion

NP is attributable to either nervous system dysfunction or neuronal damage, thus leading to abnormal pain. As a complex disorder, NP is often accompanied by abnormal gene expression [16].

The management of NP remains a challenge for clinicians. Therefore, there is an urgent need to deepen our understanding of the genetic changes behind the pathogenesis of NP to find novel potential genetic targets for NP treatment.

In recent years, bioinformatics technology has been commonly utilized to dissect the molecular mechanisms underlying NP and has revealed several relevant DEGs [17]. ATF3, JUN, and GPR151 are mainly correlated with cytokine–cytokine receptor interaction and p53 signaling in the SNI model [18]. CCL2, FCER1G, NF-*κ*B1, RAC2, and C1Q [19], p53 [20], CCL3, CTLA2B, ATF3, PLEK, and TGIF1 [21] are associated with the pathogenesis of NP.

In the present work, three gene expression datasets (GSE24982, GSE30691, and GSE63442) were integrated for RRA analysis to screen out DEGs. RRA analysis is a widely utilized tool for the integration of genome-wide gene expression data from different datasets and the identification of the key genes that are most likely to be implicated in the development of the disease under investigation [22].The DEGs identified comprise REG3B [23] and ATF3 [19, 24], which have been reported to assume a major role in the etiology of NP. After RRA analysis, enrichment analyses were conducted on 736 DEGs, which elucidated that they were mainly enriched in ion transmembrane transport [25], membrane potential regulation [26], sensory perception of pain [27], response to axon injury [28], and potassium ion transport [29]. GO and KEGG enrichment analyses uncovered that these functions were associated with the occurrence and development of NP. Additionally, complement and coagulation cascade [30], neuroactive ligand-receptor [31], and ECM-receptor interactions [32] were signaling pathways related to various functions mediated by NP. The genes associated with these pathways were also observed in our analysis. Based on the above findings, we further evaluated the roles of these genes in NP development.

WGCNA and coexpression network analysis were applied to find the hub genes involved in the pathogenesis of NP. The turquoise module that showed the highest connectivity with traits was chosen to further identify the genes in the module. Seven candidate hub genes (MKI67, VOM2R75, TJP1, EXT1, FOXP1, RNASEH2C, and EMC4) were retrieved after filtering for connectivity, GS, and MM values. To study the biological functions of these hub genes, they underwent GSEA and GSVA. The results show that most were associated with cation channel activity. Finally, MKI67, TJP1, EXT1, RNASEH2C, and EMC4 were selected as hub genes for further confirmation.

The expression of the five hub genes was determined in an SNI rat model. As reflected by RT-qPCR results, MKI67 expression was elevated, and EMC4 expression was diminished in SNI model rats compared to the sham-operated rats, which are consistent with the results of microarray analysis. There were differences in animal models and experimental conditions, and only a one-time point was selected for verification in this study, which might cause differences in the final results. Therefore, more rigorous experiments are warranted in the future for the comprehensive exploration of the role of these genes. However, our results suggest that EMC4 is a very sensitive factor and is potentially an important indicator of prognosis or prediction.

NP results from nerve injury, in which glial cell activation is one of the most prominent characteristics. Proliferation, upregulated cell surface markers and receptors, and functional changes are typical features associated with glial cell activation [33].The nuclear protein MKI67 is a well-known proliferation marker that is employed to assess cell proliferation. The number of Ki67-positive astrocytes and microglia is enhanced following peripheral nerve injury in rats, and the proliferation of glial cells may contribute to central sensitization [34]. Thus, MKI67 may participate in the pathological process of NP and may be the basis for the development and maintenance of hyperalgesia [35].

There are very few reports on the role of EMC4 and no reports on its role in NP in PubMed. EMC4 is a subunit of the endoplasmic reticulum (ER) membrane protein complex (EMC). EMC is a highly conserved oligomeric complex located on the ER membrane, which is essential for the folding and lipid transport of transmembrane proteins [36]. In all organisms evaluated, EMC destruction results in a pleiotropic phenotype. The phenotypes associated with EMC4 disruption are stress response activation in organisms and cells [37].

The ER is a pivotal organelle related to maintaining Ca^2+^ homeostasis, cell death signaling, and posttranslational modification [38]. Numerous factors, such as cellular stress (glucose deficiency and depletion of ER Ca^2+^ reserves), can trigger imbalances in the structure and function of ER, thereby leading to ER stress [39]. The ER stress response may be a crucial factor in the formation of a wide range of inflammatory diseases and neuroinflammation [40]. Nerve injury in an NP model induces an ER stress response in the DRG [41]. The repression of ER stress effectively relieves NP, which is regarded as an indicator for ER stress [42]. Therefore, we speculated that ER stress was correlated with the induction of NP. Our data show that EMC4 expression was dramatically reduced on day 14 after SNI surgery. This decrease in EMC4 expression may be associated with post-SNI pain, and ER stress may exert effects. Whether and how classic pain targets respond to ER stress or ER stress, which is alleviated, remains an active research topic. Hence, further work is needed to explore the related mechanisms.

The hub genes in NP were explored in this study. Unlike in prior studies, we did not return to the database for validation. The screened hub genes were validated in an NP rat model, which likely yields more reliable results. Relying on the reproducibility and phenotypic consistency of the animal model, we can continue to map the subsequent downstream signaling molecular pathways. Transgenic animals were also obtained through knockout or knock-in techniques to further verify whether the hub genes we screened facilitate or suppress NP.

However, our study has some limitations. The lack of sample size predetermination based on a priori power calculations and the small sample size available for analyses may obscure rare interactions, and further studies are required to dissect the relevant mechanisms.

## 5. Conclusions

EMC4 was downregulated in the SNI rat model and was a sign of NP occurrence. This finding provides novel insights into the mechanism of NP development and the associated therapeutic targets. In the future, we will ascertain the specific action mechanism of EMC4 in the development of NP to clarify its function in the pathogenesis of this condition.

## Figures and Tables

**Table 1:** Primer sequences used for qRT-PCR.

Gene	Forward primer (5′-3′)	Reverse primer (5′-3′)
MKI67	CCTGCCTCAGATGGCTCAA	GGTTCCCTGTAACTGCTCCCT
TJP1	ATGAGCGGGCTACCTTACTG	ATGCGAGCGACCTGAATG
EXT1	AACTCAAAGGAGCGGTGGG	CAGGTAATGGGAATACAGGTAGTGA
RNASEH2C	GGGAAGCAGCGTATTCACCT	GCGAAACGACACCTGTAGCC
EMC4	ACACCATTTCCATCTTCCCTACT	TTCCCAATGAGATAGACCAAGC

**Table 2:** PWT changes of rats in different groups (*n* = 6).

Group	*n*	0 (d)	3 (d)	7 (d)	10 (d)	14 (d)
Sham-con	6	18.385 ± 4.107	17.793 ± 4.207	18.190 ± 4.139	18.499 ± 3.391	19.018 ± 5.072
Sham-ips	6	18.007 ± 5.819	19.636 ± 4.659	19.393 ± 4.847	17.717 ± 4.092	16.290 ± 3.328
SNI-con	6	18.513 ± 3.356	18.551 ± 3.639	18.579 ± 3.606	17.689 ± 4.514	18.023 ± 2.874
SNI-ips	6	17.260 ± 2.859	11.671 ± 1.374*∗*	6.608 ± 3.569*∗∗∗*	5.610 ± 2.962*∗∗*	5.822 ± 2.424*∗∗∗*

Notes: *F*_group_ = 3.914, *P* = 0.006; *F*_*time*_ = 21.947, *P* *≤* *0*.*001*;  ^*∗*^, *P* < 0.05,  ^*∗∗*^, *P* < 0.01,  ^*∗∗∗*^, *P* < 0.001 compared with the Sham-ips group. Repeated measures ANOVA was used, followed by Tukey's multiple comparisons test. Data are presented as mean ± standard deviation.

**Table 3:** The expression of the hub genes detected by RT-qPCR (Mean ± standard deviation/95% CI of Mean Difference).

Group	*n*	Mki67		Tjp1		Ext1		Rnaseh2c		Emc4	
		Mean ± SD	95% CI	Mean ± SD	95% CI	Mean ± SD	95% CI	Mean ± SD	95% CI	Mean ± SD	95% CI
Sham	6	1.000 ± 0.271	1.22–2.89	1.000 ± 0.169	−0.348–0.439	1.000 ± 0.236	−0.006–0.867	1.000 ± 0.368	−0.128–0.711	1.000 ± 0.234	−0.787–0.297
SNI	6	3.053 ± 0.797*∗∗∗*		1.045 ± 0.371		1.431 ± 0.401		1.291 ± 0.269		0.458 ± 0.054 ^*∗∗*^	
*t*		5.976		0.273		2.269		1.565		−5.527	
*P*		0.001		0.793		0.053		0.151		0.002	

Notes:  ^*∗∗*^, *P* < 0.01, ^*∗∗∗*^, *P* < 0.001 compared with the Sham group. The data in the two groups were normally distributed. Student's *t*-test was carried out to compare the expression of the five hub genes. The data were expressed as mean ± standard deviation.

## Data Availability

The datasets of GSE24982, GSE30691, and GSE63442 can be downloaded from the website https://www.ncbi.nlm.nih.gov/geo/and other datasets analyzed during the current study are available from the corresponding authors upon reasonable request.

## Supplementary Materials

Supplementary Materials Supplementary Figure 1. PPI network of DEGs from RRA analysis. DEGs, differentially expressed genes; PPI, protein-protein interaction; RRA, robust rank aggregation. Supplementary Figure 2. GSEA of candidate hub genes in the GEO dataset. (A) C, (E) and (*G*) GSEA of the single candidate hub genes in GO terms. (A) VOM2R75, (C) TJP, (E) FOXP1, (G) EXT1, and(B, (D) F, and (H) GSEA of the single candidate hub genes in KEGG pathways. (B) VOM2R75, (D) TJP, (F) FOXP1, and (H) EXT1. GEO, Gene Expression Omnibus; GO, Gene Ontology; GSEA, gene set enrichment analysis. Supplementary 1. Upregulated and downregulated genes obtained by RRA analysis. Supplementary 2. Top 50 genes with the highest connectivity. Supplementary 3. Enrichment of DEGs in the turquoise module in GO and KEGG pathways. Supplementary 4.Heatmap of the top 20 upregulated and downregulated genes identified by RRA analysis. Supplementary 5. Repeated measure ANOVA followed by Tukey's multiple comparison test was used to evaluate the paw withdrawal threshold (PWT) between the Sham and SNI groups in Figure 8(a) and Table 2. Supplementary 6.Expression of the five hub genes in the Sham and SNI groups measured using RT-qPCR in Figure 8(b) and Table 3.
