# Supplementary figures and images for: Analysis of Potential Hub Genes for Neuropathic Pain Based on Differential Expression in Rat Models
Source: Pain Res Manag. 2022 Mar 3;2022:6571987. doi: 10.1155/2022/6571987 (PMC8913144; doi:10.1155/2022/6571987)

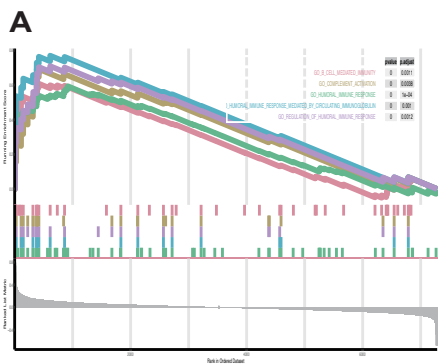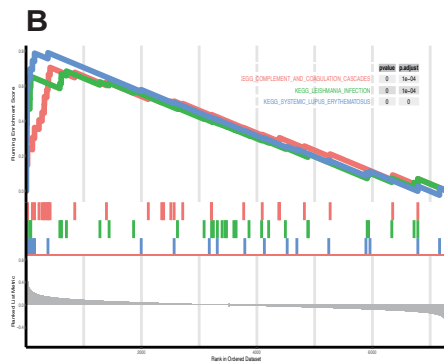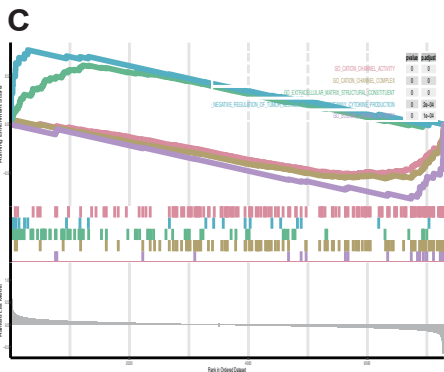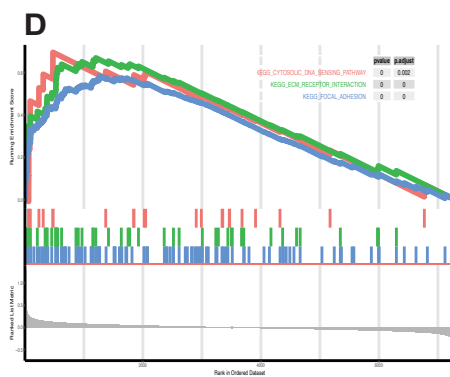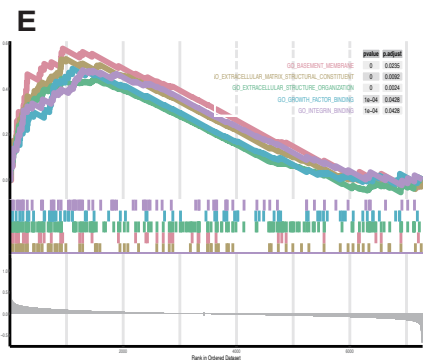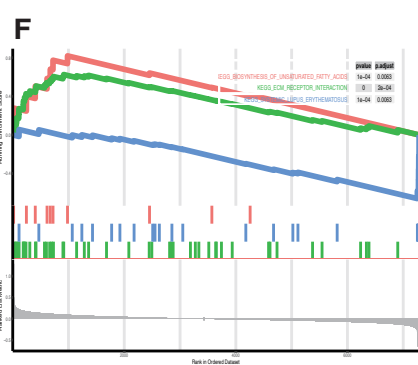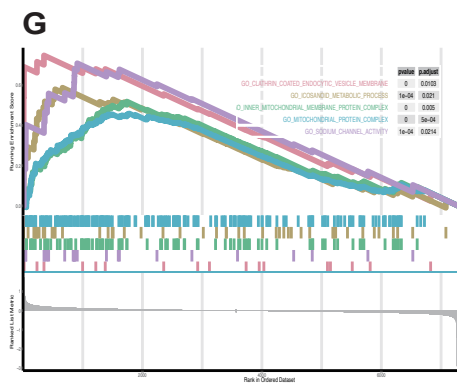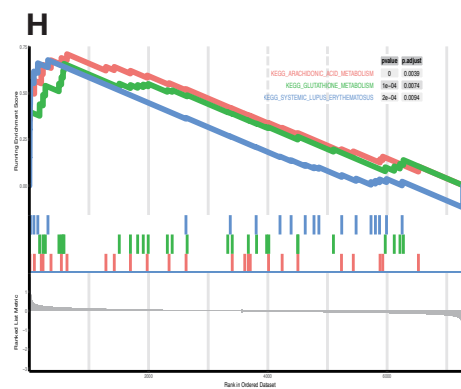

Supplement: Supplementary Materials — Supplementary Figure 1. PPI network of DEGs from RRA analysis. DEGs, differentially expressed genes; PPI, protein-protein interaction; RRA, robust rank aggregation. Supplementary Figure 2. GSEA of candidate hub genes in the GEO dataset. (A) C, (E) and (G) GSEA of the single candidate hub genes in GO terms. (A) VOM2R75, (C) TJP, (E) FOXP1, (G) EXT1, and(B, (D) F, and (H) GSEA of the single candidate hub genes in KEGG pathways. (B) VOM2R75, (D) TJP, (F) FOXP1, and (H) EXT1. GEO, Gene Expression Omnibus; GO, Gene Ontology; GSEA, gene set enrichment analysis. Supplementary 1. Upregulated and downregulated genes obtained by RRA analysis. Supplementary 2. Top 50 genes with the highest connectivity. Supplementary 3. Enrichment of DEGs in the turquoise module in GO and KEGG pathways. Supplementary 4.Heatmap of the top 20 upregulated and downregulated genes identified by RRA analysis. Supplementary 5. Repeated measure ANOVA followed by Tukey's multiple comparison test was used to evaluate the paw withdrawal threshold (PWT) between the Sham and SNI groups in Figure 8(a) and Table 2. Supplementary 6.Expression of the five hub genes in the Sham and SNI groups measured using RT-qPCR in Figure 8(b) and Table 3. [file 6571987.f1.zip › 6571987.f1/Supplement Figure 2.pdf]
